# Phenological Variation and Evolution Across Space and Time in the Scarlet Monkeyflower (*Erythranthe cardinalis*)

**DOI:** 10.1002/ece3.72752

**Published:** 2025-12-19

**Authors:** Jordan Waits, Nathanael Larson, M. C. Moazed, Ashley Regan, Marissa Strebler, Lluvia Flores‐Rentería

**Affiliations:** ^1^ Department of Biology San Diego State University San Diego California USA; ^2^ Department of Plant and Microbial Biology North Carolina State University Raleigh North Carolina USA

## Abstract

Climate change poses an existential threat to global biodiversity, especially in the California Floristic Province, where selective pressures differ across the ranges of latitudinally distributed species. Lower latitude populations face extreme heat and drought stress but may be older and more likely to harbor alleles adapted to these conditions. Conversely, poleward populations are likely younger and face a rapidly changing climate. Quantifying intraspecific variation in traits across latitudinal gradients is vital for predicting evolutionary responses to climate change. Flowering phenology, the seasonal timing and intensity of plant reproduction, varies across a species' range and is closely tied to plant strategies for dealing with drought. In this study, we combine a resurrection study and a common garden experiment at the southern edge of the scarlet monkeyflower's ( *Erythranthe cardinalis* ) range to investigate how its flowering phenology and life history have evolved in response to recent climate change. We grew seeds from the northern, central, and southern regions of its range, collected in 2010 (ancestors) and 2017 (post‐drought descendants). We measured height at first flower, rhizome production, and constructed flowering curves from June to September for full‐sibling seed families. We estimated additive genetic variance and heritability for phenological metrics and found that both decreased regionally from south to north. Southern populations began flowering later and at a larger size compared to central and northern populations, although southern descendants were shorter relative to ancestors. Conversely, northern populations produced substantially more rhizomes and began flowering earlier and shorter overall. All populations exhibited greater heritability for early‐season flowering; however, latitudinal differences were most pronounced for late‐season flowering, which shifted substantially in descendants of one central population. Northern and southern populations represent opposite phenotypic extremes regarding late‐season flowering but may exhibit increasingly greater synchrony in early‐season flowering.

## Graphical abstract

Climate change is altering ecological dynamics, particularly in species with latitudinal range variation. This study investigates how the scarlet monkeyflower's flowering phenology has evolved in response to recent droughts in California. We find that southern populations are flowering later and larger, while northern populations flower earlier and smaller, with only one central population displaying an evolutionary shift in late‐season flowering. Our findings reveal greater genetic variance in the south and examine how this has shaped reproductive strategy shifts in the wake of climate change.

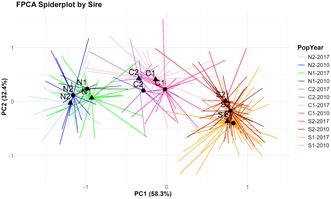

## 1 Introduction

Climate change poses an existential threat to plant populations around the globe, specifically in biodiversity hotspots such as California (Bellard et al. 2014). As the atmosphere warms, the ideal climatic niche for many species will shift poleward in latitude or upwards in elevation, where conditions are more temperate (Parmesan and Yohe 2003), although global patterns in climate‐induced range shifts are more diverse (Lenoir and Svenning 2015). Species that can keep pace with these climatic shifts or adapt to emerging climates are expected to persist through time (Hickling et al. 2006). Populations at the leading (poleward) and trailing (equatorial) edges of ranges face different selective pressures. As interannual temperature increases are greatest at higher latitudes (Loarie et al. 2009; IPCC 2023), the persistence of leading‐edge populations depends on gene flow of beneficial alleles from trailing‐edge populations (Davis and Shaw 2001; Aitken et al. 2008). Studies have shown that such gene flow can occur frequently in nature, especially in species capable of long‐distance pollen dispersal (Paul et al. 2011; Kremer et al. 2012), and that it can promote local adaptation to climate change (Aitken and Whitlock 2013; Wadgymar et al. 2022).

Trailing‐edge populations, specifically those near the arid horse latitudes, are expected to represent a phenotypic extreme along their species' latitudinal gradient that is better adapted to heat and drought stress (Halbritter et al. 2015). Therefore, there are few populations from which gene flow could rescue trailing populations under decline from future climatic extremes (Carlson et al. 2014). Instead, their evolutionary rescue and subsequent restoration of population size depend on high amounts of standing genetic diversity to ensure alleles for adaptive phenotypes increase in frequency, which can occur in populations declining gradually due to protracted stress (Bell 2013).

Many adaptive traits are influenced by multiple genes (polygenic), in that the phenotype of that trait represents the additive effects of each segregating allele. This additive genetic variance in phenotype represents the available genetic material that natural selection can act upon (Barrett and Schluter 2008). Estimates of additive genetic variance are crucial for determining how heritable a trait is across generations as well as how strongly it is being selected for, via the Breeder's equation (Conner et al. 2003). Early research into genetic variation across species ranges estimated that the greatest diversity would be found in the center and gradually decrease towards fragmented, peripheral populations that were also highly differentiated (Eckert et al. 2008). However, this *center–periphery* hypothesis was adapted to postulate that the greatest genetic diversity is found in the oldest populations, rather than the most central, and that these ancient populations are often found near trailing edges (Pironon et al. 2017). Although lower latitudes harbor trailing populations today, they provided climatically stable refugia to ancient populations between glacial periods, thus supplying much of the genetic material for postglacial poleward recolonization (Hewitt 2000; Hampe and Petit 2005). For species whose trailing margins are highly fragmented, genetic diversity likely reaches its maximum in admixture zones where these highly differentiated populations converge (Petit et al. 2003; Pironon et al. 2017). It is at these convergence zones near trailing margins and niche edges where we would expect to see a proportional increase in both the heritability and additive genetic variance for polygenic traits (Conner et al. 2003; Pennington et al. 2021). Quantifying variation in phenotypes under selection across demographic regions is a crucial step in modeling species persistence under future climate scenarios.

As climate change intensifies drought across mid‐latitudes, specifically in California (Cook et al. 2018), populations are under stronger selection for traits that enhance drought tolerance or escape (Kooyers 2015). Phenology, which is the timing of seasonal or cyclic biological events, plays a central role in these adaptive responses, with substantial shifts documented in insects (Renner and Zohner 2018), animals (Cohen et al. 2018), and especially plants (D. W. Inouye 2008; Collins et al. 2021). In plants, flowering time is highly heritable (Brachi et al. 2011) and polygenic (Zan and Carlborg 2019) and reflects key fitness trade‐offs: flowering too early can risk frost damage (D. W. Inouye 2008) or pollinator mismatch (Straka and Starzomski 2014), while flowering too late can truncate seed maturation (Kudo and Hirao 2006). Flowering and setting seed early in the season before the onset of drought conditions is a primary example of a drought “escape” strategy (Shavrukov et al. 2017) and is becoming more frequent as spring temperatures rise interannually (Anderson et al. 2012; Petrauski et al. 2019). On the other hand, drought “avoidance” involves increased growth of roots and vegetative structures that improve water use efficiency (Seleiman et al. 2021), which allows them to flower later into the season (Kenney et al. 2014). Different drought strategies, such as faster growth, earlier flowering, and greater seed production (escape) versus slower, water‐efficient growth, later flowering, and increased root development (avoidance), are often mutually exclusive (Kooyers et al. 2014), genetically linked (Fletcher et al. 2015), and maintained through antagonistic pleiotropy (Mckay et al. 2003), although environmental cues can plastically induce either strategy (Des Marais et al. 2013). In clonal species such as *E. cardinalis* , rhizome production reflects investment in belowground persistence and may facilitate drought avoidance. However, previous studies have reported conflicting patterns of rhizome production across species and environments (Zwicke et al. 2015; Nelson et al. 2021), suggesting that its relationship with flowering time and drought strategy remains unresolved. Obtaining empirical measurements of phenological and vegetative phenotypes in real world populations will be crucial for accurately modeling adaptation, plasticity, and range shifts under future climate change (Valladares et al. 2014). However, because flowering phenology is a continuous process over an entire year, the metric used to describe temporal and spatial trends in flowering time can cause results and inferences to vary markedly.

The majority of phenological research focuses on point estimates of phenological firsts like day of first flower because it is under selection by climate change, varies across populations, and can be easily measured in the field and in the greenhouse (Anderson et al. 2012; Petrauski et al. 2019; Anstett et al. 2021; Vtipil and Sheth 2020). Relatively less attention has been given to intraspecific variation in the shape of entire flowering curves (Collins et al. 2021), despite their potential to provide deeper insights into how selection shapes population‐level phenological traits (Forrest and Miller‐Rushing 2010) and their ecological consequences—particularly regarding drought strategies, functional diversity, and the temporal availability of phenological niches (Albert et al. 2012; Vitt et al. 2020). A long‐term study of phenological evolution in a subalpine plant community found that basing assessments of phenological shifts on a single measurement of flowering (first, peak, or last flowering) underestimated the number of responsive species by 18%–38% (CaraDonna et al. 2014). This highlights the need to interpret phenological traits over their complete temporal distribution, in order to capture interactions with other traits, avoid erroneously attributing them to a certain strategy, and to ensure future conservation decisions are ecologically and evolutionarily relevant.

In order to test whether differences in flowering time between populations have an evolutionary or genetic basis, as opposed to being strictly plastic, they need to be grown together under identical conditions. Common gardens are an established means to quantify geographic trends in phenotype (Clausen et al. 1948; Sandquist and Ehleringer 1997; Ramírez‐Valiente et al. 2022) and additive genetic variance, when the relatedness of propagules is known (de Villemereuil et al. 2015). As common gardens measure how traits change across space, resurrection studies measure how they change across time by growing seeds collected from the same population in different years (Franks et al. 2014). The scarlet monkeyflower ( *Erythranthe cardinalis* ) extends from Baja California to southern Oregon and is a model system for studying how variation in phenotype is distributed across latitudinal gradients and how it is genetically controlled (Schemske and Bradshaw Jr 1999; Wu et al. 2008). Previous common garden experiments with *E. cardinalis* showed that populations from the southern range margin exhibited the greatest responses to selection for flowering time, suggesting they harbor greater genetic diversity than higher latitude populations (Sheth and Angert 2016). Southern populations of *E. cardinalis* also tended to germinate and grow faster (Muir and Angert 2017) with demographic studies suggesting that trailing‐edge populations exhibit a more annualized life‐history strategy indicative of drought “escape” (Sheth and Angert 2018). Recently, resurrection studies in a greenhouse and growth chambers compared an ancestral cohort collected in 2010 and a descendent cohort in 2017, respectively before and after a period of prolonged drought in California (Vtipil and Sheth 2020; Wooliver et al. 2020). There was no change between generations for flowering time (Vtipil and Sheth 2020) and a narrower thermal performance breadth for only one southern population (Wooliver et al. 2020), which also experienced the most severe drought between 2010 and 2017. Although greenhouse common gardens are informative, their inability to create an environment that perfectly simulates natural conditions limits the scope of inferences that can be made from them. In order to directly predict how these different populations may respond to future climate change, we repeated this resurrection experiment (Vtipil and Sheth 2020) in an ecological reserve at the trailing margin of the scarlet monkeyflower's range. We specifically emphasize evolution over the entire distribution of a flowering curve, as this may illuminate trends not captured by phenological firsts alone.

In this study, we aim to describe how traits relevant for adapting to drought have evolved across the latitudinal range of a widely distributed species. By growing all cohorts in natural conditions near the trailing range edge, we can directly estimate how additive genetic variance in ancestral, predrought populations influenced evolutionary responses in descendants for coping with heat and drought stress. Under a combined common garden and resurrection study framework, we pursue the following objectives: (1) To quantify shifts in phenological curves between pre‐ and postdrought cohorts of *Erythranthe cardinalis* across latitudinally distributed populations, with a particular focus on early‐season flowering. (2) To evaluate whether southern populations harbor greater additive genetic variance for phenological traits as displayed through artificial selection (Sheth and Angert 2016) and predicted by a *trailing‐leading* model for age and genetic diversity (Hampe and Petit 2005; Pironon et al. 2017). (3) To examine how different populations make evolutionary tradeoffs between flowering phenology and vegetative traits, such as rhizome production and height at first flower. We expect that a more pronounced influence of drought and higher additive genetic variance at the trailing range edge of *Erythranthe cardinalis* will cause descendants from southern populations to exhibit the greatest phenological shift toward early flowering. For these same reasons, we anticipate southern plants will be taller at first flower (Vtipil and Sheth 2020) and produce less rhizomes than northern plants (Nelson et al. 2021), indicative of a drought escape strategy. Describing how drought traits are genetically maintained across the range of *E. cardinalis* will have far‐reaching applications for the conservation of California native plants and latitudinally distributed biodiversity in general.

## 2 Materials and Methods

### 2.1 Study Species and Crossing Design

**FIGURE 1:**
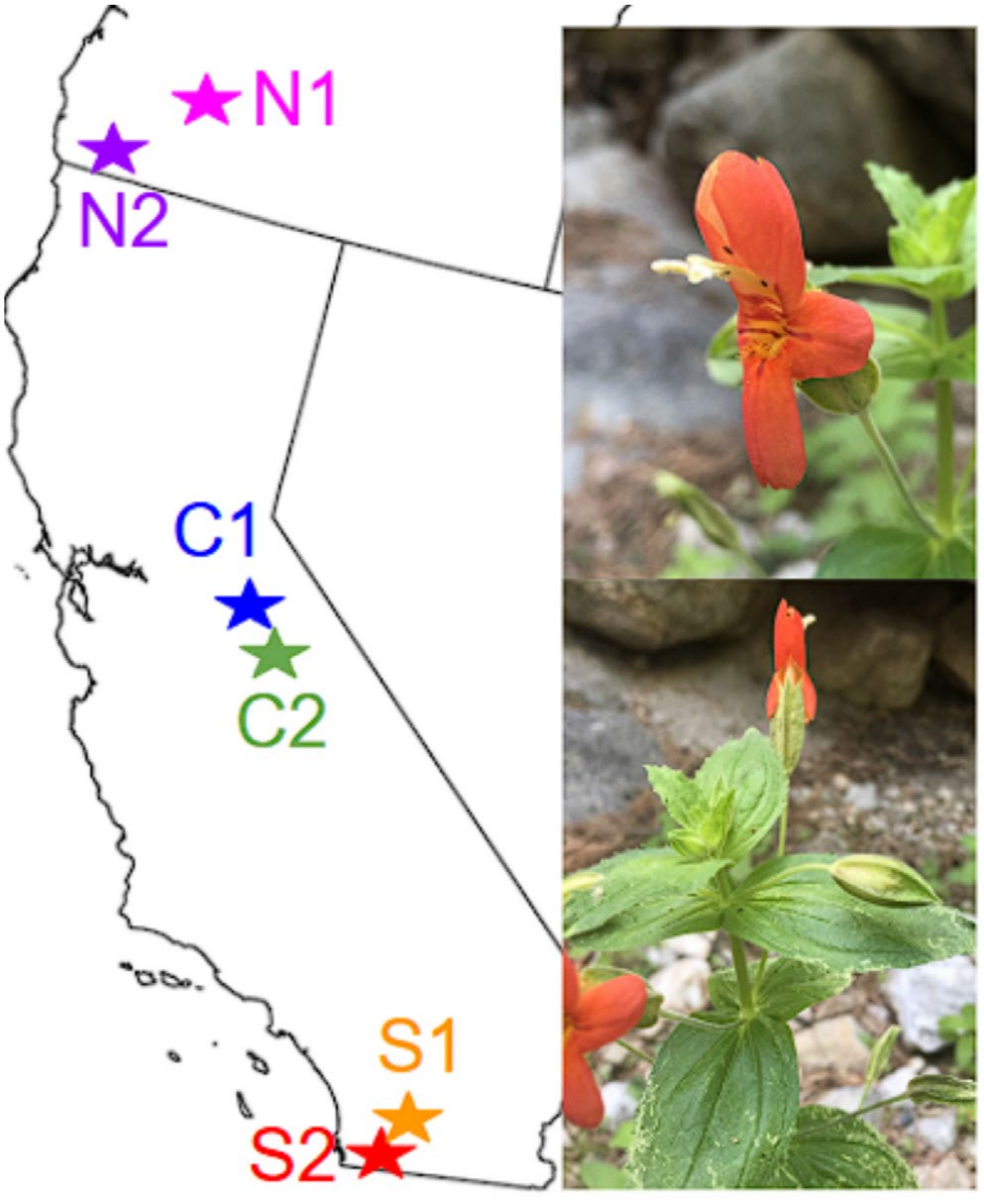
Simple map showing where study populations were sourced from as well as photographs of *Erythranthe cardinalis* , the focal species. Specific coordinates for each location can be found in Sheth and Angert (2016).

*Erythranthe cardinalis* , or the scarlet monkeyflower, is a perennial herb that spreads and regenerates via a rhizome. It inhabits seepage and riparian areas from southern Oregon to northern Baja California, including the Coast and Sierra Nevada ranges but absent in the Central Valley. Past studies on the species have described foundational knowledge of local adaptation, geographic range limits, and responses to climate change (Angert and Schemske 2005; Angert et al. 2011; Paul et al. 2011; Wooliver et al. 2020). Before this experiment, seeds were collected from 57 to 216 individuals in two northern‐edge (N1 and N2), two central (C1 and C2), and two southern‐edge (S1 and S2) populations (Figure 1) in Fall 2010 (ancestral cohort) and 2017 (descendant cohort) as described in Sheth and Angert (2016). Between the two collection years, California experienced a pronounced drought that was felt with differing intensity by the six study populations (Vtipil and Sheth 2020), except in one southern population that actually experienced reduced drought intensity. In general, drought conditions increase in severity from north to south. Between 2018 and 2019, a crossing design was adapted (Falconer and Mackay 1996; Wang 2007) by the Sheth lab in the greenhouses at North Carolina State University to establish a framework to measure additive genetic variance. For both cohorts, maternal seed families were created by randomly crossing wild‐collected individuals within each population, resulting in 40–101 full‐sibling families per population. This followed a nested paternal half‐sibling design, in which 8–33 unique sires were crossed with 3–5 unique dames per combination of population and year. Obtaining seed families with known parentage allows us to accurately estimate how traits are inherited across generations and quantify how the traits of the parent influence the traits of the offspring.

### 2.2 Greenhouse Procedure

In late March of 2023, a total of 7776 seeds representing all population‐year combinations were germinated at San Diego State University's Alvarado Greenhouse. A range of 3–12 seeds (mean = 6) were planted from each of the 1137 seed families, with roughly three times as many 2010 individuals as 2017 individuals. Due to restrictions on garden size in the ecological reserve, this imbalance in sample size was chosen to focus statistical power on estimating additive genetic variance in ancestor cohorts and establishing a phenotypic baseline from which to compare descendants. Seedlings were grown under controlled temperatures (27°C day/20°C night) in 72‐cell trays using a layered mixture of SunGro Sunshine Mix #4 Professional Growing Mix and Quikrete Premium Play Sand. Trays were misted until germination, then bottom‐watered exclusively, and rotated weekly to minimize microclimate variation. After 6 weeks, total germination had passed 90% and seedlings were of sufficient size to be transplanted.

### 2.3 Common Garden Experiment

The field common garden was established at the Santa Margarita River Ecological Reserve (latitude: 33°26′18″ N, longitude: 117°10′36″ W, elevation: 263 m), representing the southernmost of three gardens meant to simulate the trailing edge, core, and leading edge of the species' range. The 820 m^2^ site, located adjacent to a tributary stream with naturally occurring *E. cardinalis* , was leveled and enclosed by a 1.8 m mammal‐exclusion fence lined with buried hardware cloth and a silt screen to deter herbivory. To loosen the clay soil and remove persistent weeds, the garden was turned over with a gas‐powered tiller once in late November 2022 and again, after heavy rainfall, in February 2023, finally being covered with gray landscape fabric. The garden was divided into 10 long blocks separated by 0.5 m walkways, consisting of four rows per block. Each plant in a row was positioned 0.3 m apart and every other row was staggered by 0.15 m to offset rows and reduce crowding. A central PVC irrigation line that bisected the garden extended drip tubing along each of the 80 rows, ensuring uniform water availability to all plants. A total of 5468 seedlings were transplanted (see Table 1) between May 8 and May 24, 2023 in a randomized design to ensure spatial separation of related individuals. Watering frequency was gradually reduced through the growing season to simulate the natural summer dry‐down typical of ephemeral stream habitats, shifting from four to five times per week from May to July down to once per week by late August.

**TABLE 1:** Exact numbers of ancestors, descendants, dams, and sires planted into the common garden for each population.

Population	Ancestors	Descendants	Dams	Sires
S2	926	293	247	50
S1	898	221	258	58
C2	421	182	96	20
C1	809	229	233	53
N2	648	214	167	41
N1	332	225	133	33
Total	4034	1364	1134	255

### 2.4 Data Collection and Phenological Curve Metrics (Objective 1)

Beginning on June 20th, 2023 and ending on September 6th, 2023, censuses for flowering in the garden were taken roughly every 4 days, for a total of 18 censuses. For all planting positions, a “V,” “F,” or “D” was recorded, respectively indicating the plant in that position was either “Vegetative,” “Flowering,” or “Dead.” Plants in “F” had at least one active flower in anthesis, plants in “V” had no flowers in anthesis (wilted flowers with nonfunctioning stigmas were not counted as flowering), and plants that were “D” had no living aboveground biomass. If a plant that previously had not begun flowering clearly had its first flower and dropped it before a given census, then it would be recorded as an “F” for that census.

In order to explore how different populations and years vary across the entire range of their flowering season, percentages of individuals in flower were calculated at each time point. First, the original multinomial count data (V, F, D) was transformed into binary data for “flowering” and “not flowering.” The binary count data were then calculated as a proportion in flower for each maternal line (each Dam), representing the probability of flowering on a given day for any offspring from that unique full‐sibling family (2–12 individuals/full‐sib family, mean = 5). This resulted in 1134 total full‐sib families nested within 255 half‐sib families, each of which could be represented as a curve over the range of Day 171 to Day 249 (of the year). These data will form the basis of all phenological analyses going forward.

**FIGURE 2:**
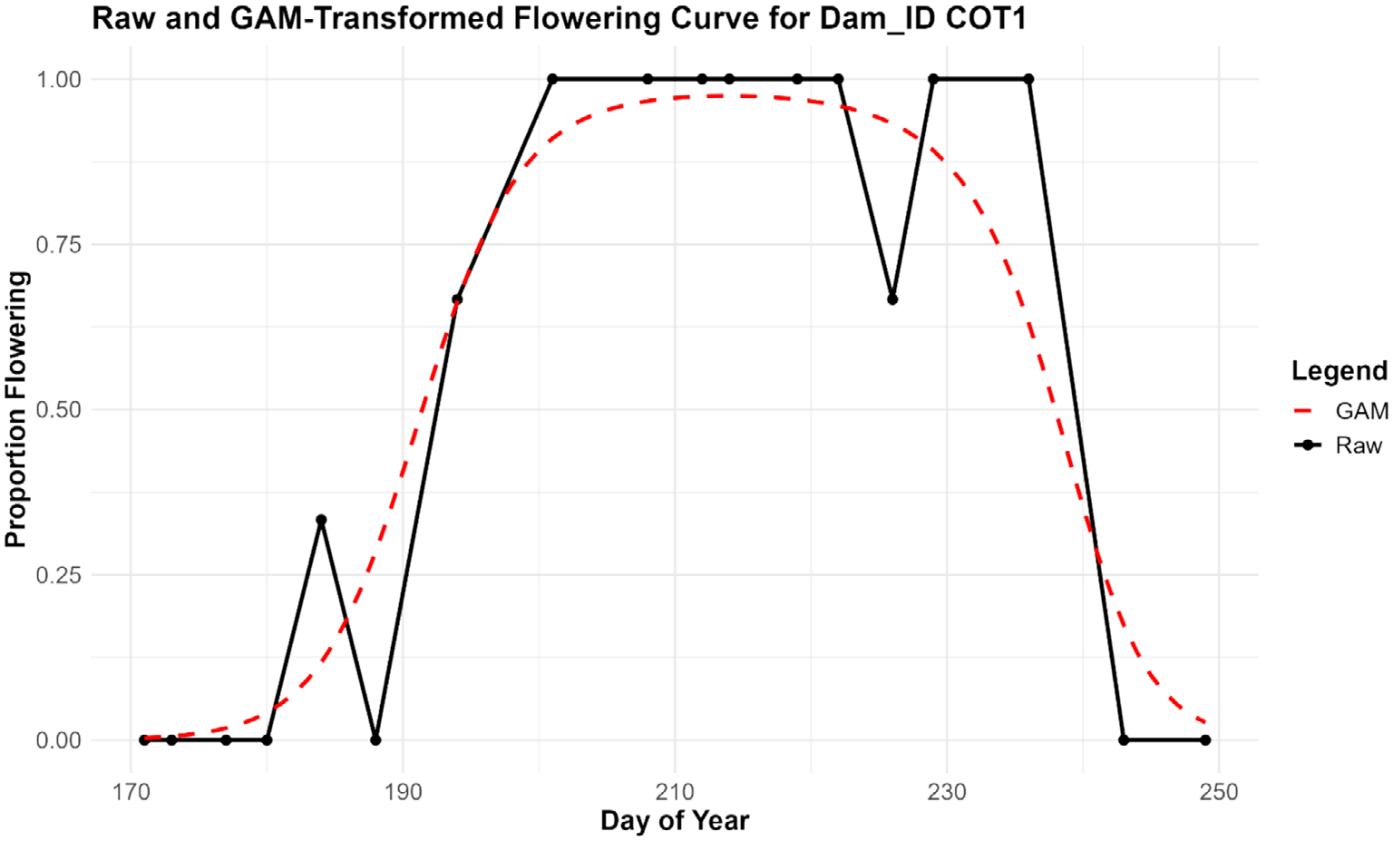
Figure of total flowering curve for a single full‐sibling maternal family (dam). The black solid line represents the curve created by the original data and the dashed red line represents the curve created by the GAM‐transformed data. The generalized additive model evaluated proportions in flower using a logit link function for each day across the study range from Day 171 to Day 249.

**FIGURE 3:**
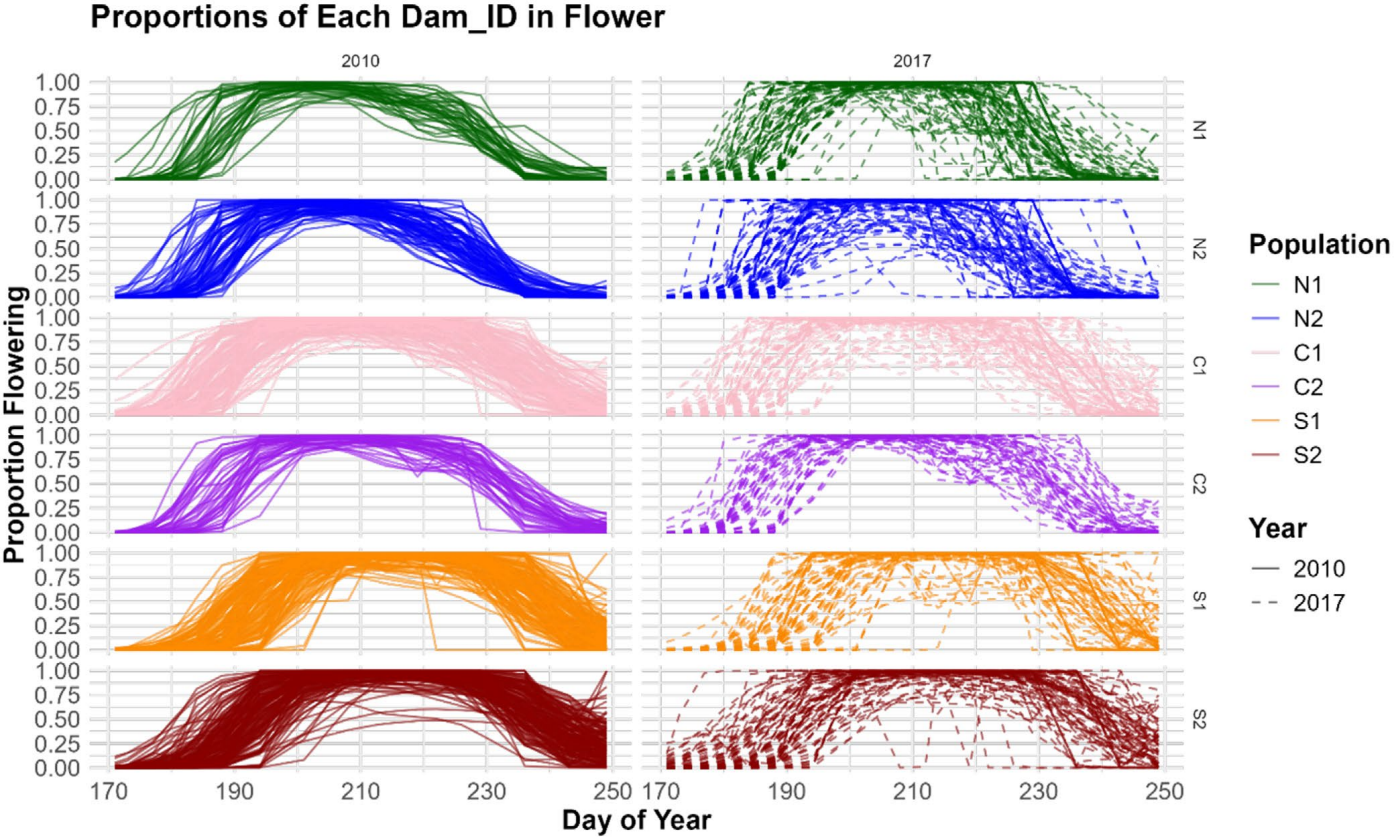
Figure of total flowering curves for each maternal line from each population and year. Populations are color coded and year is denoted by a solid line (2010) and a dashed line (2017). Data were transformed with a generalized additive model that evaluated proportions in flower using a logit link function from each day across the study range from Day 171 to Day 249.

Due to the often small sample size for each maternal line, curves could rapidly change from 0% to 100% flowering between census days. In order to smooth these curves enough to estimate certain phenological metrics, each dam (full‐sibling family) was fitted with a simple Generalized Additive Model (GAM). We used the gam function from the “mgcv” package in R and a binomial family with logit link to estimate flowering proportions across all 79 days from Day 171 to Day 249. Differences in the flowering curve constructed from the original data and from the GAM‐transformed data are exemplified for a single dam family in Figure 2 and for all families across all populations in Figure 3. We then integrated the area under the GAM‐estimated curves of each maternal line (full‐sib family) to find days where each curve reached 5%, 25%, 50%, 75%, and 95% area under the curve (AUC), respectively corresponding to season start, peak start, peak center, peak end, and season end.

Estimating flowering metrics with curve integration tangibly quantifies the total reproductive effort exerted by each maternal line in the garden, rather than assuming all curves reached a certain percent threshold (i.e., day of 75% flowering). In this way, curve integration reduces error introduced by uneven sample sizes among full‐ and half‐sibling families and stochastic events influencing individual curves (i.e., insect damage, edaphic factors). We fit a linear mixed‐effects model (LMM) using the lmer function from the “lme4” package (Bates et al. 2015) to test for differences in flowering metrics across populations and years. For each metric, we fit a model with fixed effects for population, year, and their interaction and a random effect for sire family to account for statistically nonindependent observations from shared sires within populations. We used Type III analysis of variance with Satterthwaite‐adjusted degrees of freedom (via the “lmerTest” package; Kuznetsova et al. 2017) to assess the significance of fixed effects. Estimated marginal means of flowering metrics were extracted using the emmeans package (Lenth 2023) and pairwise contrasts between populations were adjusted for multiple comparisons using Tukey's method. For metrics that differed between cohorts, we performed six a priori pairwise comparisons between 2010 and 2017 within each population, applying the Holm adjustment to control the family‐wise error rate. In contrast, a standard ANOVA with Tukey's post hoc test would involve 66 pairwise comparisons and treat all observations as independent, disregarding genetic relatedness and thereby inflating Type I error due to pseudoreplication (Bolker et al. 2009; Harrison et al. 2018).

### 2.5 Functional Principal Components Analysis (Objective 1)

Functional Principal Components Analysis (FPCA) was used to capture continuous variation in the entire shape of flowering curves, offering insights beyond point estimates or discrete moments in time. Unlike first or last flowering day measures, FPCA provides a comprehensive analysis of temporal dynamics, revealing how early‐season flowering behavior is associated with late‐season patterns, as well as covariation in timing, amplitude, and duration. Due to the chronological relatedness of each census day, a simple PCA is unsuitable because it would treat each day as an independent vector of values. FPCA, on the other hand, does account for chronology and treats each curve as a replicate or observation. As a result, the FPCA outputs eigenfunctions that represent the principal “modes of variation” of the data (Hurley et al. 2014), rather than eigenvectors as is the case with PCA. Using the unaltered curves for each maternal line, we used a nonparametric method called “principal components analysis through the conditional expectation” (PACE) through the FPCA function and R “fdapace” package. PACE is a useful tool in this case because it interpolates values between sparse census days.

We specified only two Principal Components for this test, which were obtained for each maternal line and explained 90.5% of the variance in flowering curves. As with flowering metrics, a linear mixed model with fixed effects of population, year, and their interaction, and a random effect of sire family on Principal Component scores. A Multivariate Analysis of Variance (MANOVA) tested the effects of year, population, and sire on the two Principal Components jointly, with pairwise contrasts between cohorts conducted using permutation MANOVAs (*n* = 999). The MANOVA summary statistic, Pillai's trace, is robust to uneven sample sizes and multicollinearity (Krzanowski 1988), which is inevitable given the chronological nature of our dependent variable. To visualize how curves differ across each FPC, twice the square root of the principal component variance was added and subtracted to the total mean flowering function and plotted against it (Ramsay and Silverman 2002).

### 2.6 Genetic Variance at Trailing and Leading Range Edges (Objective 2)

By growing plants from different populations and years together in a common garden where each plant experiences the same environment, we can directly estimate how phenotypic differences are attributable to genotype rather than environment. In this study, we estimated additive genetic variance ($V_{a}$) and narrow‐sense heritability ($h^{2}$) for phenological metrics (Season Start, Peak Start, Peak Center, Peak End, and Season End) and Functional Principal Component scores (PC1 and PC2) using a Bayesian animal model implemented in the *MCMCglmm* package in R (Hadfield 2010). For each ancestral population (2010 cohort), we fit univariate mixed‐effects models to partition phenotypic variance into genetic and residual components. Each model was structured as:

$$Y=\mu +V_{\text{sire}}+V_{R}$$

where Y is the phenotype of a given individual, μ is the overall mean of the trait across the population, $V_{\text{sire}}$ is the random effect representing the between‐sire genetic variance, and $V_{R}$ is the residual variance introduced by nonadditive genetic effects (environment, dominance, and epistasis). We specified weakly informative inverse‐Gamma priors for the sire and variance components, which allow estimates to be primarily informed by the data over assumptions. Because the dimensionality of FPCs is already reduced, a slightly less informative prior was used for FPCs (*ν* = 0.01) than phenological metrics (*ν* = 0.05). To ensure all estimates had an Effective Sample Size greater than 1000, each model was run for 150,000 iterations with a 30,000‐iteration burn‐in period and a thinning interval of 25 to reduce autocorrelation. Chain convergence and mixing were assessed through visual inspection of trace plots and stationarity was confirmed through the Heidelberger and Welch diagnostic (Heidelberger and Welch 1983). In the case of half‐siblings, the variance in phenotype between sire families ($V_{\text{sire}}$) is equal to the covariance within families. Additive genetic variance ($V_{a}$) can then be estimated by dividing $V_{\text{sire}}$ by two times the coefficient of kinship, defined as the probability that two individuals share two alleles that are identical by descent (for half‐siblings, ⅛) (Falconer and Mackay 1996; Wang 2007). For each of our models, $V_{a}$ was calculated as four times $V_{\text{sire}}$, which was then divided by the sum of $V_{a}$ and $V_{R}$, or total phenotypic variance ($V_{P}$) to obtain the narrow‐sense heritability ($h^{2}$), according to the equation below:

$$4\times \mathrm{var}\left(\text{sirex},\text{sirey},\text{sirez}\right)=\frac{\mathrm{cov}\left(\text{sibx},\text{siby},\text{sibz}\right)}{2F_{\mathrm{xyz}}}=V_{a}=h^{2}\times V_{P}$$

Posterior distributions for $V_{a}$, $V_{R}$, and $h^{2}$ were summarized by calculating their mean estimates and 95% highest posterior density (HPD) intervals, representing the range within which the true parameter value falls with 95% probability.

### 2.7 Relationship Between Rhizomes and Phenology (Objective 3)

When each plant had its first recorded flower, the lengths of its three tallest stems were measured from their meristems to the soil surface at the base of the plant and averaged to obtain height at first flower (HFF). In March 2024, a subsample of 50 plants was haphazardly removed from the garden to assess rhizome production in each of the populations. An effort was made to choose plants nearby each other to minimize random effects of planting position location, although high end‐of‐year mortality among northern populations limited options. Each plant was dug up, pruned of any stems above ~1 in. from the soil surface, and washed to remove the soil. With only the rhizomes and root ball present, the plants were dried and weighed, and this weight was divided by the total number of rhizomes to obtain an average rhizome weight (ARW) per plant. The effect of population, year, and their interaction on height at first flower (HFF) was analyzed using the same linear mixed model as previous variables. However, because HFF was measured for each individual, rather than aggregated within maternal families, the random effect term in the model uses dam rather than sire family. Because we lacked adequate sample size across years for rhizome data, only the effect of population was included in a simple linear model for ARW, number of rhizomes, and total rhizome biomass. The relationship between HFF and ARW among populations was described using Pearson correlation with the cor.test function in R.

## 3 Results

### 3.1 Phenological Curve Metrics (Objective 1)

Linear mixed models revealed a highly significant effect of population on all flowering metrics (Table 2) and a strong latitudinal trend showing later flowering in the south and earlier flowering in the north. Pairwise differences between populations grow stronger later in the season, with Season Start being undifferentiable among the four central and northern populations and Season End being statistically distinct among all populations, except N1 and N2, which were undifferentiable for all metrics (Table 3). The two southern populations were distinct from each other for all metrics except Peak Center and Peak End, while central populations were only distinct for Peak End and Season End. The interaction of population and year insignificantly affected all flowering metrics; however, there was a significant effect of year alone on Season End. Pairwise contrasts of each population between 2010 and 2017 reveal descendant cohorts of C1 and N1 both reached Season End about 2 days earlier than their ancestors, although this difference is only marginally significant in N1. This pattern is reflected across populations, with descendant cohorts reaching Season End 1.32 days earlier than ancestors, on average.

**TABLE 2:** Summary statistics for linear mixed models of the five phenological metrics.

Metric	Effect	Variance	SD	Marginal *R* ^2^	Conditional *R* ^2^	Sum Sq.	Mean Sq.	NumDF	DenDF	*F* value	*p*
Season start	Sire_ID	4.455	2.111	0.239	0.411	—	—	—	—	—	—
Season start	Residual	15.250	3.905	—	—	—	—	—	—	—	—
Season start	Population	—	—	—	—	2905.567	581.113	5	240.056	38.105	0.000
Season start	Year	—	—	—	—	1.422	1.422	1	238.838	0.093	0.760
Season start	Population:Year	—	—	—	—	101.938	20.388	5	240.056	1.337	0.249
Peak start	Sire_ID	2.413	1.554	0.408	0.523	—	—	—	—	—	—
Peak start	Residual	10.014	3.165	—	—	—	—	—	—	—	—
Peak start	Population	—	—	—	—	4293.436	858.687	5	238.380	85.748	0.000
Peak start	Year	—	—	—	—	8.494	8.494	1	237.035	0.848	0.358
Peak start	Population:Year	—	—	—	—	72.471	14.494	5	238.380	1.447	0.208
Peak center	Sire_ID	1.043	1.021	0.554	0.600	—	—	—	—	—	—
Peak center	Residual	8.997	2.999	—	—	—	—	—	—	—	—
Peak center	Population	—	—	—	—	8504.984	1700.997	5	235.973	189.068	0.000
Peak center	Year	—	—	—	—	15.249	15.249	1	234.158	1.695	0.194
Peak center	Population:Year	—	—	—	—	55.123	11.025	5	235.973	1.225	0.298
Peak end	Sire_ID	0.218	0.467	0.559	0.567	—	—	—	—	—	—
Peak end	Residual	12.436	3.527	—	—	—	—	—	—	—	—
Peak end	Population	—	—	—	—	15,259.693	3051.939	5	228.356	245.406	0.000
Peak end	Year	—	—	—	—	24.256	24.256	1	225.916	1.950	0.164
Peak end	Population:Year	—	—	—	—	20.488	4.098	5	228.356	0.329	0.895
Season end	Sire_ID	0.114	0.338	0.491	0.494	—	—	—	—	—	—
Season end	Residual	19.697	4.438	—	—	—	—	—	—	—	—
Season end	Population	—	—	—	—	17,513.324	3502.665	5	225.696	177.824	0.000
Season end	Year	—	—	—	—	391.481	391.481	1	223.168	19.875	0.000
Season end	Population:Year	—	—	—	—	71.973	14.395	5	225.696	0.731	0.601

**TABLE 3:** Pairwise comparisons of ancestor and descendant cohorts for the metrics Season Start, Peak Center, and Season End.

Metric	Population	Estimate (2017–2010)	SE	dF	*t*‐ratio	*p*	*p* (Holm)	95% CI (upper: lower)
Season start	S1	0.309	0.753	250.590	0.411	0.682	1.000	−1.174: 1.792
Season start	S2	−1.359	0.808	219.642	−1.681	0.094	0.565	−2.952: 0.235
Season start	C1	−0.330	0.778	247.353	−0.424	0.672	1.000	−1.862: 1.203
Season start	C2	−0.574	1.243	225.521	−0.462	0.645	1.000	−3.022: 1.875
Season start	N1	1.638	1.152	247.137	1.421	0.157	0.783	−0.632: 3.907
Season start	N2	1.034	0.914	255.646	1.131	0.259	1.000	−0.767: 2.834
Peak center	S1	−0.295	0.472	255.617	−0.625	0.533	1.000	−1.224: 0.634
Peak center	S2	−0.760	0.498	210.131	−1.527	0.128	0.641	−1.742: 0.221
Peak center	C1	−1.048	0.487	251.431	−2.153	0.032	0.194	−2.007: −0.089
Peak center	C2	−0.731	0.768	217.724	−0.952	0.342	1.000	−2.245: 0.782
Peak center	N1	0.532	0.720	247.045	0.739	0.460	1.000	−0.886: 1.951
Peak center	N2	0.395	0.574	262.103	0.688	0.492	1.000	−0.735: 1.526
Season end	S1	−1.126	0.578	264.350	−1.950	0.052	0.209	−2.263: 0.011
Season end	S2	−0.771	0.595	198.734	−1.296	0.197	0.393	−1.945: 0.402
Season end	C1	−2.007	0.595	258.835	−3.374	0.001	0.005	−3.178: −0.836
Season end	C2	−0.654	0.922	207.838	−0.710	0.479	0.479	−2.472: 1.163
Season end	N1	−2.117	0.878	245.441	−2.413	0.017	0.083	−3.846: −0.389
Season end	N2	−1.246	0.705	271.196	−1.768	0.078	0.235	−2.633: 0.142

**FIGURE 4:**
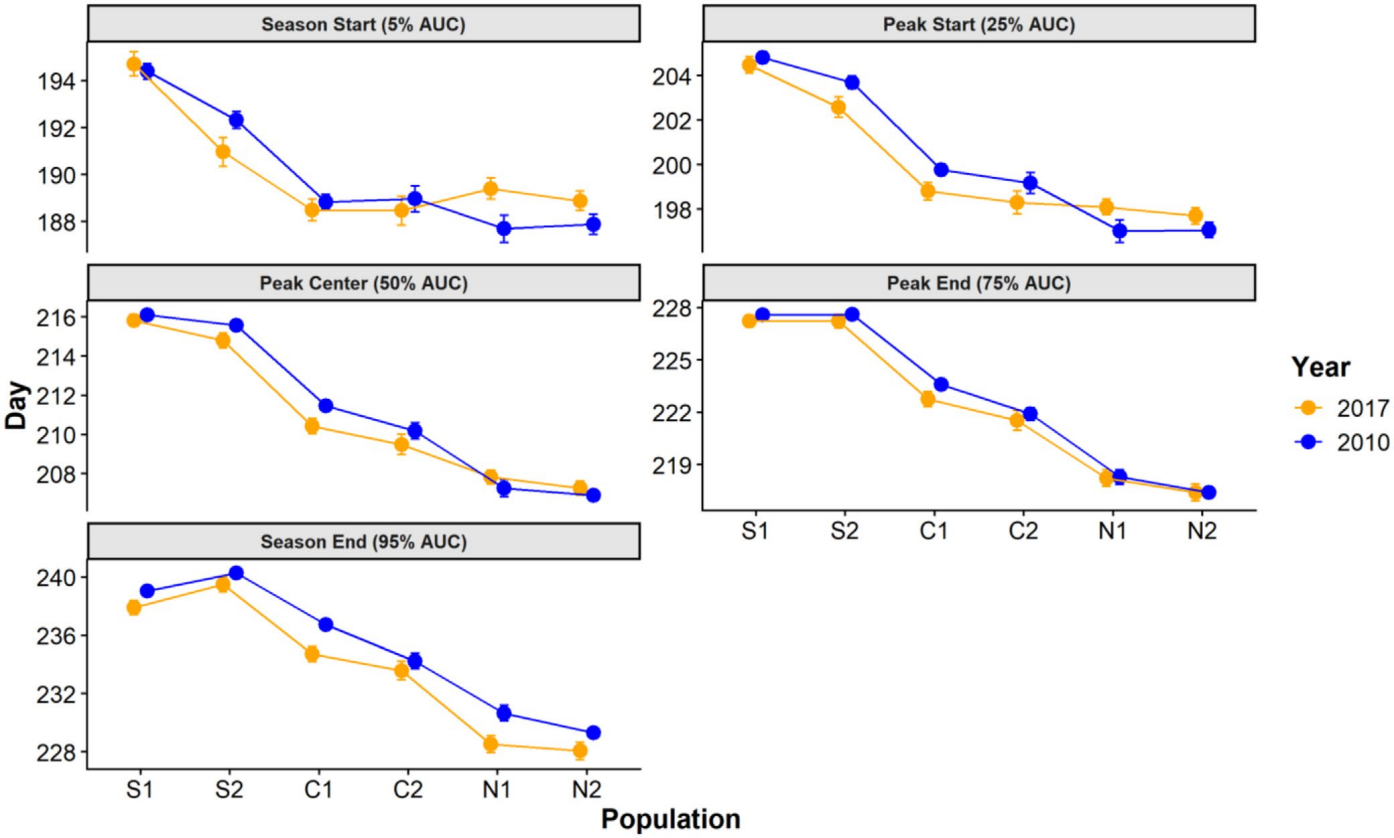
Faceted line plot for each of the five phenological metrics: Season Start, Peak Start, Peak Center, Peak End, and Season End. Respectively these represent the day on which each curve reached 5%, 25%, 50%, 75%, and 95% area under the curve (AUC) as calculated through integration. For each population, descendants are in orange and ancestors are in blue, with points representing the cohort mean along with standard error bars.

Despite the absence of significant differences, pairwise tables for Season Start and Peak Center are displayed along with Season End (Table 3) to highlight patterns and effect sizes. Peak Center appears to be the inflection point where C1 descendants begin to deviate from their ancestors, with the two cohorts virtually indistinguishable in earlier metrics and increasingly distinct in later metrics. Visualizations of flowering metrics (Figure 4) imply that S2 descendants reached Season Start earlier than their ancestors while N1 and N2 descendants reached it later, suggesting evolutionary change in opposite directions. However, these differences are insignificant when adjusted for multiple comparisons and only marginally significant in S2 using preadjusted *p* values. Including the random effect for sire improved model fit (*R* ^2^) for all metrics and yielded lower AIC than simpler linear models without the random effect. Variance attributable to the random effect of sire family, relative to residual variance, was highest for Season Start and decreased gradually in later metrics, ultimately contributing so little variance in Season End that model fit barely changed (Table 2). While the ratio of sire variance to residual variance in this example does imply a pattern in additive genetic variance, it is essential to note that this is not part of our calculation of genetic variance because it includes both year cohorts.

### 3.2 Functional Principal Components Analysis (Objective 1)

**FIGURE 5:**
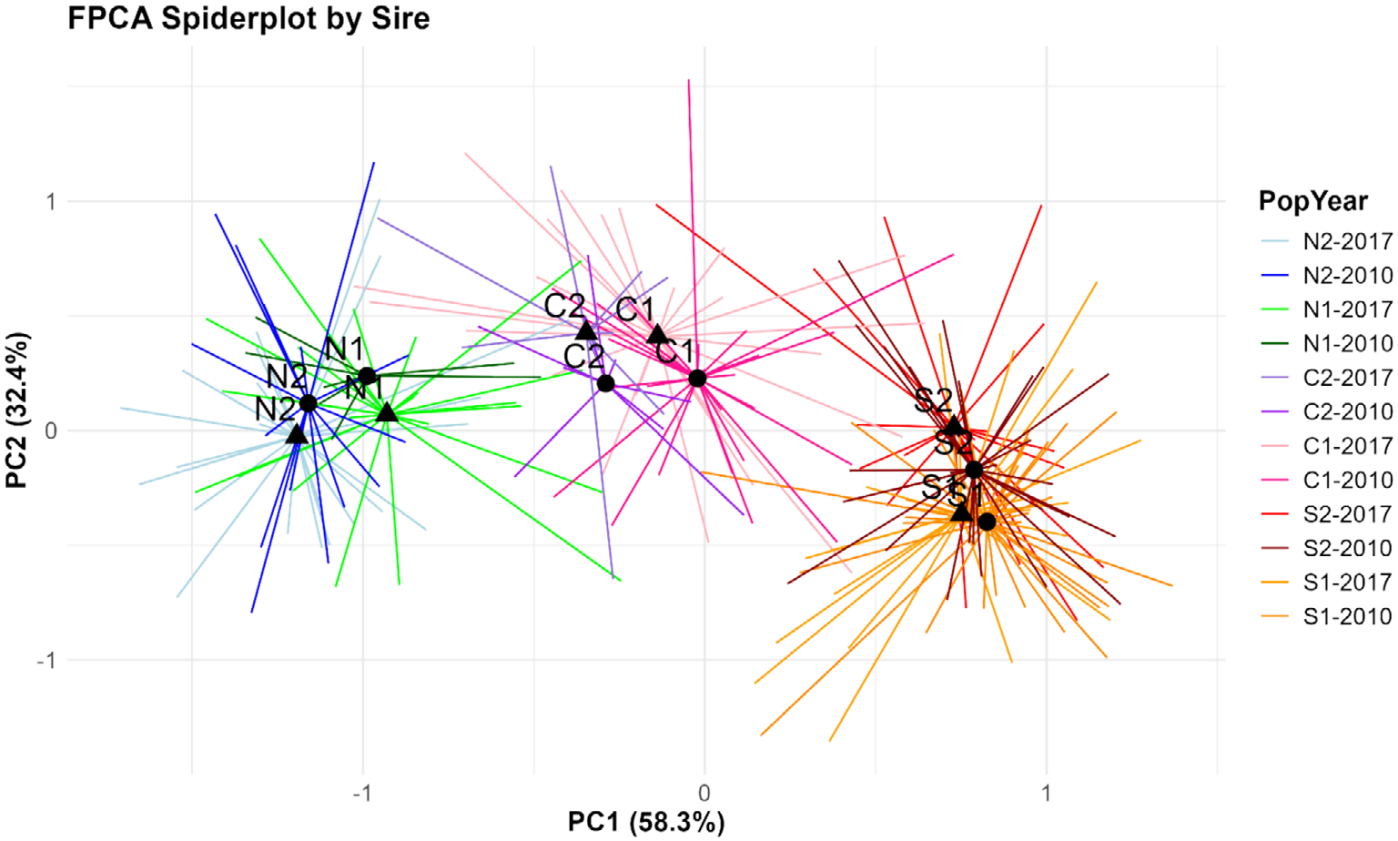
Scatter plot of Functional Principal Component Scores colored by PopYear. Each PopYear centroid is labeled by population; triangles are the 2017 cohort while circles are the 2010 cohort. Proportion of variance explained by both principal components is provided in the axis labels. Each point represents maternal families averaged over their respective sire. Points are connected to their respective centroid by a line colored by PopYear.

**FIGURE 6:**
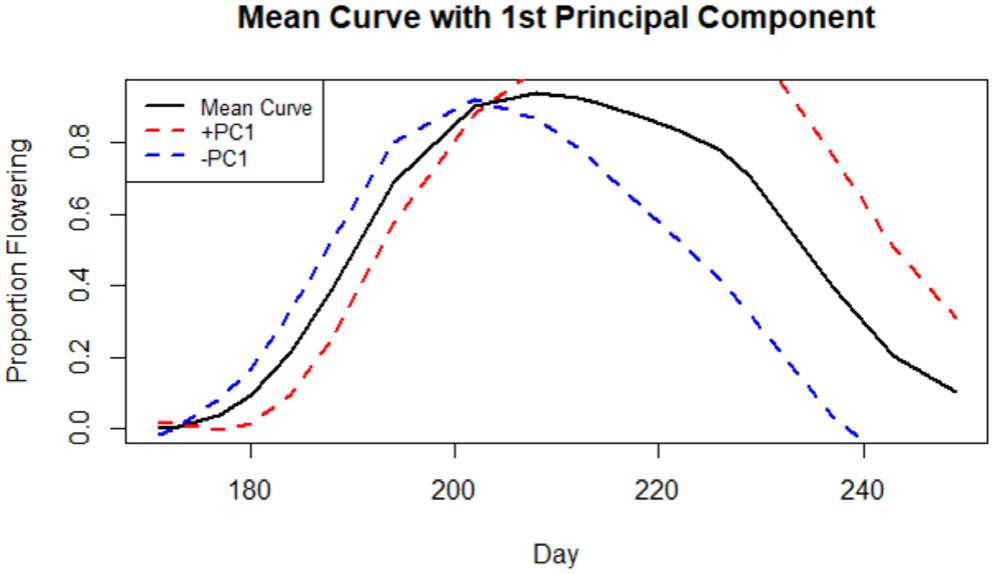
Figure showing the two dominant modes of variation for the first principal component (proportion of variance = 58.3%). The mean line for all plants is shown in black. The mean line for plants on the positive end of PC1 is shown in red and the mean line for those on the negative end of PC1 in blue.

Our FPCA accounted for a total of 90.7% of the variance in the flowering curves of maternal lines (PC1 = 58.3%, PC2 = 32.4%). The MANOVA found significant effects of year, population, and sire, which respectively had Pillai's trace values of 0.118, 0.745, and 0.577, indicating population and sire introduced 74% and 58% of the variation into the principal components (Table 4). Pairwise comparisons of all cohorts highlighted a marginally significant change in C1 descendants (*F*_2,230_ = 2.75, *R* ^2^ = 0.023, *p* = 0.088), shifting about −0.12 units along PC1 and +0.18 units along PC2 (Figure 5). Plotting the variance of PC1 against the total mean flowering function (Figure 6) reveals entire curves shift to be later with increasing positivity along PC1. In addition, more positive curves exhibit a pronounced late‐season peak in flowering and continue flowering past the study time frame. A linear mixed model revealed a highly significant effect of population on PC1 and an insignificant effect of year and its interaction with population. A strong latitudinal trend manifested along the axis of PC1, with northern populations inhabiting the negative end and southern populations on the positive end (Figure 5). All population pairs differed significantly, or marginally with N1 and N2 (+0.218 ± 0.083, *t* (255) = 2.617, *p* = 0.097), except the two southern populations, which were almost identical (+0.03 ± 0.06, *t* (227) = 0.493, *p* = 0.996).

**TABLE 4:** Summary statistics for MANOVA of functional principal components.

Source of variation	dF	Pillai's trace	Approx. *F* value	Num dF	Denom dF	*p*
Population	1	0.11847	58.998	2	878	< 2.2e‐16
Year	5	0.74489	104.334	10	1758	< 2.2e‐16
Pop:Year	248	0.57746	1.439	496	1758	8.09e‐08
Residuals	879					

**FIGURE 7:**
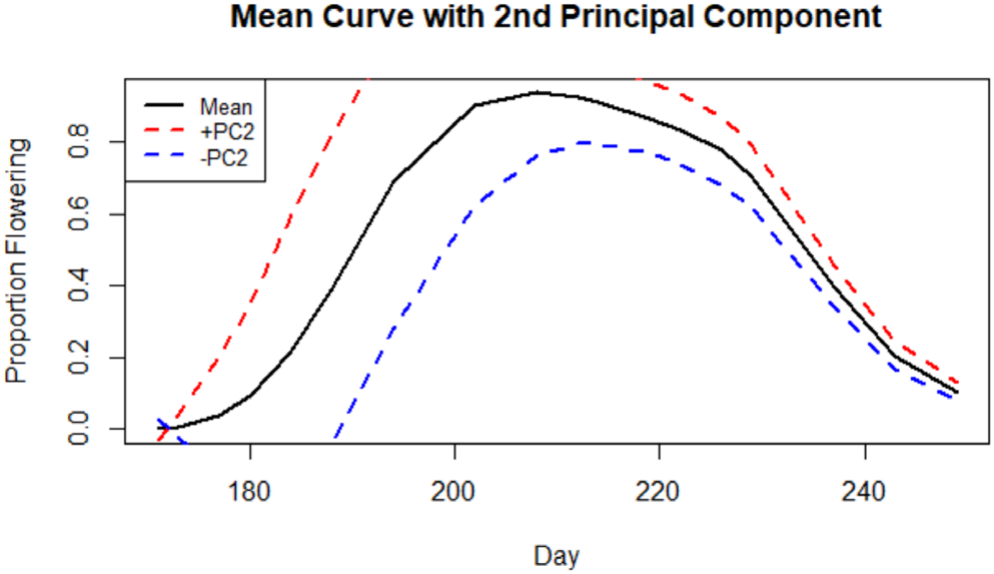
Figure showing the two dominant modes of variation for the second principal component (proportion of variance = 32.4%). The mean line for all plants is shown in black. The mean line for plants on the positive end of PC2 is shown in red and the mean line for those on the negative end of PC2 in blue.

Visualizing how curves differ along the axis of PC2 (Figure 7) reveals the component primarily describes variance in early‐season flowering, with curves on the positive end exhibiting an earlier season start and more rapid ascent to peak flowering. As with PC1, the linear mixed model for PC2 reported a highly significant effect of population, although there is no clear latitudinal trend. The most negative values of the axis are families from S1, which were statistically distinct from each of the five other populations. Families from the two central populations generally held the most positive values, although they were only distinct from the southern populations and in the pairing of C1 and N2. Together, N2, N1, and S2 clustered around the mid‐values of PC2 and were statistically very similar. Although the effect of year and its interaction with population on PC2 is insignificant (Table 5), cohorts from different years differ along PC2 visibly more than they do along PC1. The greatest change was seen in C2, S2, and C1, which each increased about 0.19 units, followed by N1, which decreased by about 0.16 units, mirroring the earlier trend of descendants from S2 and N1 changing in opposite directions.

**TABLE 5:** Summary statistics for linear mixed models of functional principal components.

Metric	Effect	Variance	SD	Marginal *R* ^2^	Conditional *R* ^2^	Sum sq.	Mean sq.	NumDF	DenDF	*F* value	*p*
PC1	Sire_ID	0.000	0.000	0.574	0.574	—	—	—	—	—	—
PC1	Residual	0.441	0.664	—	—	—	—	—	—	—	—
PC1	Population	—	—	—	—	615.170	123.034	5	1122.000	278.837	0.000
PC1	Year	—	—	—	—	0.508	0.508	1	1122.000	1.150	0.284
PC1	Population:Year	—	—	—	—	0.593	0.119	5	1122.000	0.269	0.930
PC2	Sire_ID	0.091	0.301	0.120	0.269	—	—	—	—	—	—
PC2	Residual	0.445	0.667	—	—	—	—	—	—	—	—
PC2	Population	—	—	—	—	38.725	7.745	5	236.277	17.398	0.000
PC2	Year	—	—	—	—	0.349	0.349	1	234.808	0.785	0.377
PC2	Population:Year	—	—	—	—	2.787	0.557	5	236.277	1.252	0.286

### 3.3 Genetic Variance at Trailing and Leading Range Edges (Objective 2)

Estimates of additive genetic variance ($V_{a}$) and heritability ($h^{2}$) in ancestor cohorts generally began at their maximum at Season Start and descended sharply after Peak Start (Table 6). However, C2 exhibited little change in heritability across metrics and N1 began Season Start at its minimum heritability and reached its maximum at Peak End. In decreasing order, heritability for Season Start was greatest in N2 (0.76 95% CI: 0.55, 0.92), C1 (0.61 95% CI: 0.36, 0.84), S1 (0.50 95% CI: 0.22, 0.75), and S2 (0.47 95% CI: 0.17, 0.74). This trend in heritability continues until C2 surpasses S2 at Peak Center and N1 surpasses all other populations at Peak End. Heritability for Season End was highest in S1 (0.25 95% CI: 0.00, 0.55), followed by N1 (0.22 95% CI: 0.00, 0.66) and C2 (0.21 95% CI: 0.00, 0.65). Most populations had consistently similar total phenotypic variance ($V_{P}$) for each metric, except N2 had noticeably lower $V_{P}$ for Peak End and Season End than other populations and N1 had lower $V_{P}$ for Season Start and Peak Start.

**TABLE 6:** Estimated mean additive genetic variance ($V_{a}$), residual variance ($V_{R}$), and narrow‐sense heritability ($h^{2}$) with lower and upper 95% confidence intervals for each ancestral population (2010 cohort) and phenological metric, based on MCMCglmm analysis.

Metric	Population	$V_{a}$ (95% CI)	$V_{R}$ (95% CI)	$h^{2}$ (95% CI)
Season start	S1	16.78 (2.31, 33.82)	14.88 (11.44, 18.96)	0.50 (0.22, 0.75)
Season start	S2	16.85 (0.84, 33.73)	16.86 (12.57, 21.42)	0.47 (0.17, 0.74)
Season start	C1	17.28 (3.93, 34.10)	9.73 (7.10, 12.52)	0.61 (0.36, 0.84)
Season start	C2	6.87 (0.03, 25.76)	13.29 (7.65, 19.96)	0.26 (0.00, 0.69)
Season start	N1	2.47 (0.01, 9.52)	12.19 (6.72, 18.50)	0.13 (0.00, 0.46)
Season start	N2	26.94 (5.77, 55.71)	7.19 (4.65, 10.15)	0.76 (0.55, 0.92)
Peak start	S1	9.62 (1.18, 19.24)	8.98 (6.86, 11.42)	0.49 (0.21, 0.75)
Peak start	S2	8.11 (0.04, 17.61)	11.20 (8.42, 14.21)	0.39 (0.01, 0.64)
Peak start	C1	9.10 (0.15, 18.31)	6.30 (4.63, 8.21)	0.55 (0.27, 0.83)
Peak start	C2	6.51 (0.02, 22.22)	9.05 (5.21, 13.48)	0.32 (0.00, 0.74)
Peak start	N1	2.17 (0.02, 8.39)	9.49 (5.50, 14.45)	0.15 (0.00, 0.49)
Peak start	N2	16.50 (1.57, 35.00)	5.38 (3.52, 7.52)	0.71 (0.47, 0.92)
Peak center	S1	4.15 (0.03, 9.57)	7.23 (5.45, 9.09)	0.33 (0.01, 0.60)
Peak center	S2	3.49 (0.02, 9.18)	8.24 (6.31, 10.45)	0.27 (0.00, 0.55)
Peak center	C1	3.75 (0.02, 8.92)	5.92 (4.39, 7.61)	0.35 (0.01, 0.63)
Peak center	C2	4.40 (0.02, 15.45)	6.75 (3.94, 9.98)	0.30 (0.00, 0.72)
Peak center	N1	3.21 (0.02, 12.04)	6.81 (3.78, 10.34)	0.24 (0.00, 0.67)
Peak center	N2	4.30 (0.03, 11.79)	4.74 (3.03, 6.69)	0.40 (0.01, 0.74)
Peak end	S1	3.12 (0.02, 8.58)	8.58 (6.54, 10.73)	0.24 (0.00, 0.52)
Peak end	S2	2.01 (0.02, 5.89)	7.96 (6.26, 9.98)	0.18 (0.00, 0.44)
Peak end	C1	1.44 (0.01, 4.95)	8.86 (6.74, 11.21)	0.12 (0.00, 0.37)
Peak end	C2	2.45 (0.02, 9.11)	5.74 (3.20, 8.22)	0.23 (0.00, 0.63)
Peak end	N1	6.23 (0.02, 21.38)	5.96 (3.10, 9.27)	0.38 (0.00, 0.81)
Peak end	N2	1.25 (0.02, 4.50)	5.40 (3.70, 7.53)	0.16 (0.00, 0.48)
Season end	S1	5.21 (0.02, 14.51)	13.31 (10.15, 16.66)	0.25 (0.00, 0.55)
Season end	S2	2.61 (0.02, 8.42)	11.14 (8.64, 13.89)	0.17 (0.00, 0.45)
Season end	C1	1.77 (0.02, 6.28)	13.84 (10.65, 17.40)	0.10 (0.00, 0.32)
Season end	C2	4.79 (0.02, 19.33)	12.28 (7.47, 18.23)	0.21 (0.00, 0.65)
Season end	N1	4.47 (0.02, 17.99)	10.53 (5.98, 16.15)	0.22 (0.00, 0.66)
Season end	N2	1.50 (0.02, 5.51)	8.75 (5.97, 12.01)	0.12 (0.00, 0.41)

For the two principal components, additive genetic variance and heritability was consistently higher for PC2 than PC1 (Table 7). In descending order, heritability was greatest for PC1 in N1 (0.60 95% CI: 0.17, 0.95), C2 (0.35 95% CI: 0.03, 0.72), and S1 (0.24 95% CI: 0.02, 0.50). For PC2, the greatest heritability was seen in N2 (0.80 95% CI: 0.63, 0.94), followed by S1 (0.46 95% CI: 0.18, 0.73), C1 (0.43 95% CI: 0.09, 0.71), and S2 (0.40 95% CI: 0.07, 0.66), which are notably very similar to the trends seen in heritability for Season Start. Total phenotypic variance ($V_{P}$) for each metric was consistent across populations, except in N1 and N2 who respectively had substantially higher $V_{a}$ and heritability for PC1 and PC2 than other populations.

**TABLE 7:** Estimated mean additive genetic variance ($V_{a}$), residual variance ($V_{R}$), and narrow‐sense heritability ($h^{2}$) with lower and upper 95% confidence intervals for each ancestral population (2010 cohort) and principal component (PC) score from functional principal components analysis (FPCA), based on MCMCglmm analysis.

Metric	Population	$V_{a}$ (95% CI)	$V_{R}$ (95% CI)	$h^{2}$ (95% CI)
PC1	S1	0.12 (0.01, 0.30)	0.33 (0.25, 0.41)	0.24 (0.02, 0.50)
PC1	S2	0.07 (0.00, 0.19)	0.28 (0.22, 0.35)	0.19 (0.02, 0.42)
PC1	C1	0.09 (0.00, 0.23)	0.36 (0.27, 0.45)	0.18 (0.01, 0.41)
PC1	C2	0.16 (0.01, 0.48)	0.21 (0.13, 0.31)	0.35 (0.03, 0.72)
PC1	N1	0.37 (0.01, 1.05)	0.16 (0.08, 0.25)	0.60 (0.17, 0.95)
PC1	N2	0.06 (0.00, 0.17)	0.20 (0.13, 0.27)	0.21 (0.02, 0.48)
PC2	S1	0.28 (0.03, 0.56)	0.29 (0.22, 0.36)	0.46 (0.18, 0.73)
PC2	S2	0.30 (0.01, 0.65)	0.40 (0.31, 0.51)	0.40 (0.07, 0.66)
PC2	C1	0.33 (0.01, 0.73)	0.37 (0.27, 0.48)	0.43 (0.09, 0.71)
PC2	C2	0.31 (0.00, 0.99)	0.38 (0.22, 0.56)	0.36 (0.02, 0.75)
PC2	N1	0.11 (0.00, 0.39)	0.43 (0.25, 0.65)	0.17 (0.01, 0.47)
PC2	N2	1.21 (0.31, 2.43)	0.25 (0.16, 0.36)	0.80 (0.63, 0.94)

### 3.4 Relationship Between Rhizomes and Phenology (Objective 3)

**FIGURE 8:**
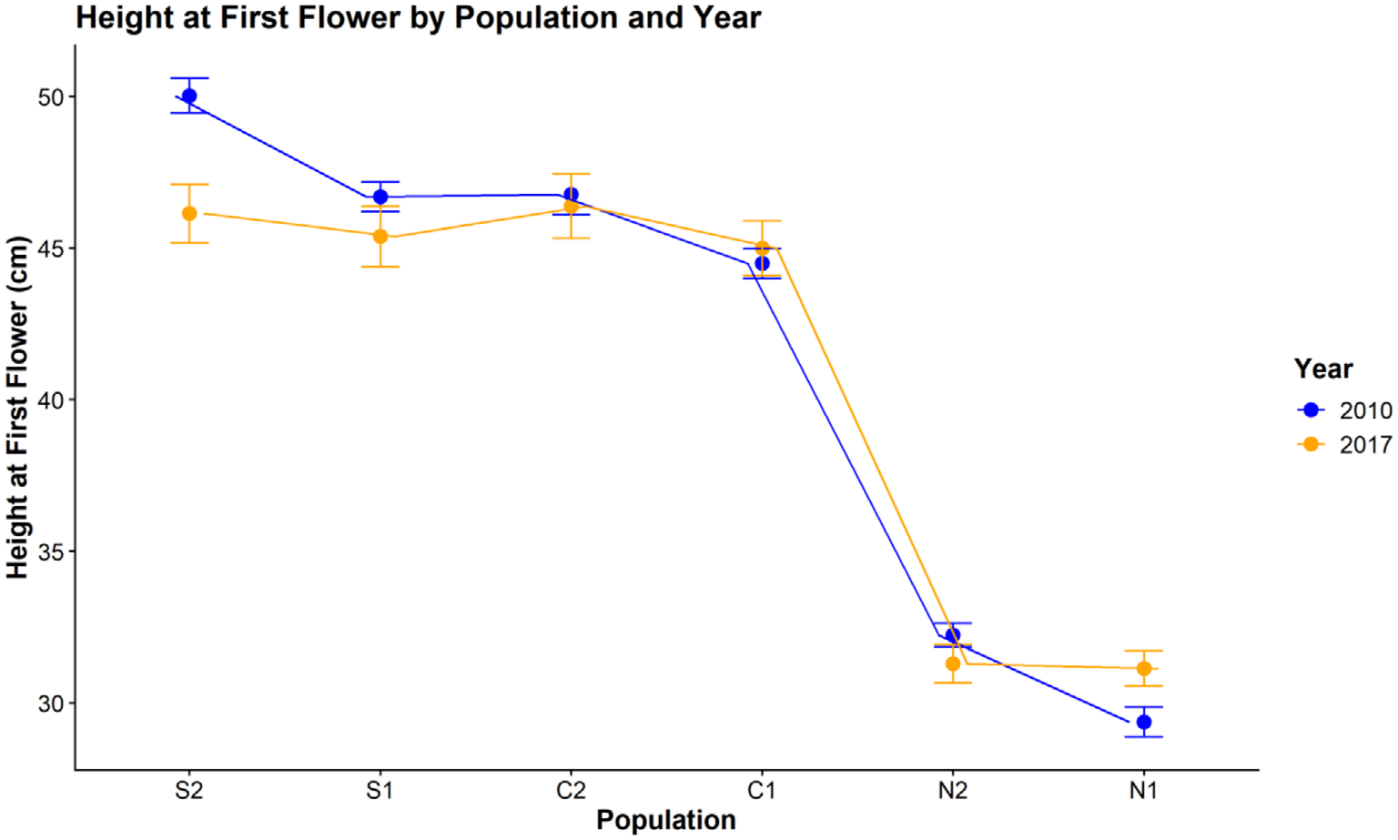
Line plot of height at first flower for each population and year. For each population, descendants are in orange and ancestors are in blue, with points representing the cohort mean along with standard error bars.

**FIGURE 9:**
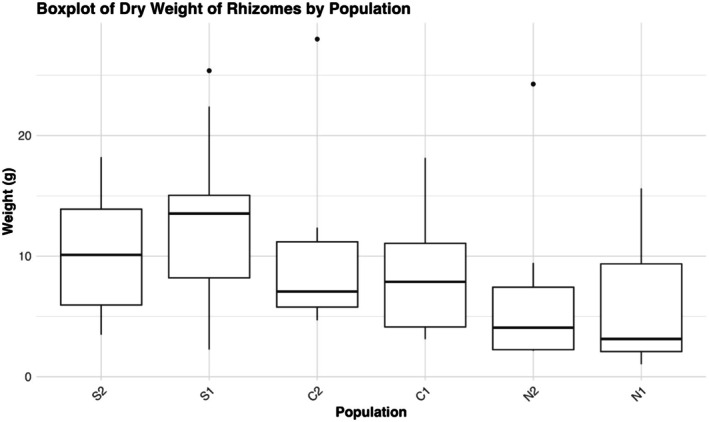
Boxplot showing the dry weight of the root ball containing rhizomes of plants from each population. Prior to drying and weighing, we removed all of the soil, stems, and leaves, and most of the roots.

**FIGURE 10:**
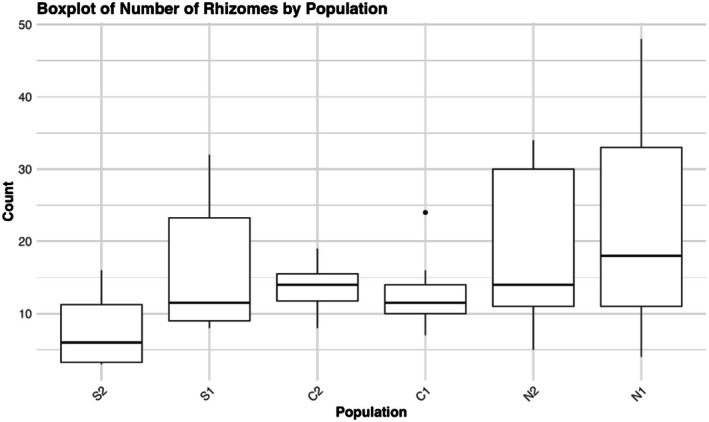
Boxplot showing the number of rhizomes counted for plants from each population. Rhizomes were counted before drying and weighing the root ball.

**FIGURE 11:**
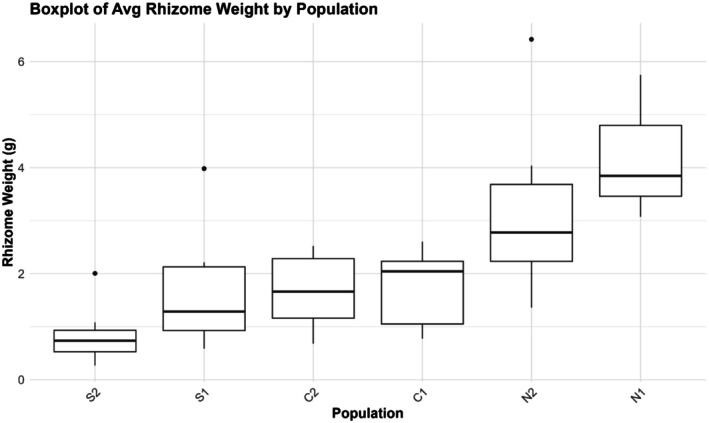
Boxplot showing the average rhizome weight (ARW) of plants from each population. ARW was calculated by dividing the total weight of the rhizomes and root ball by the total number of rhizomes.

**FIGURE 12:**
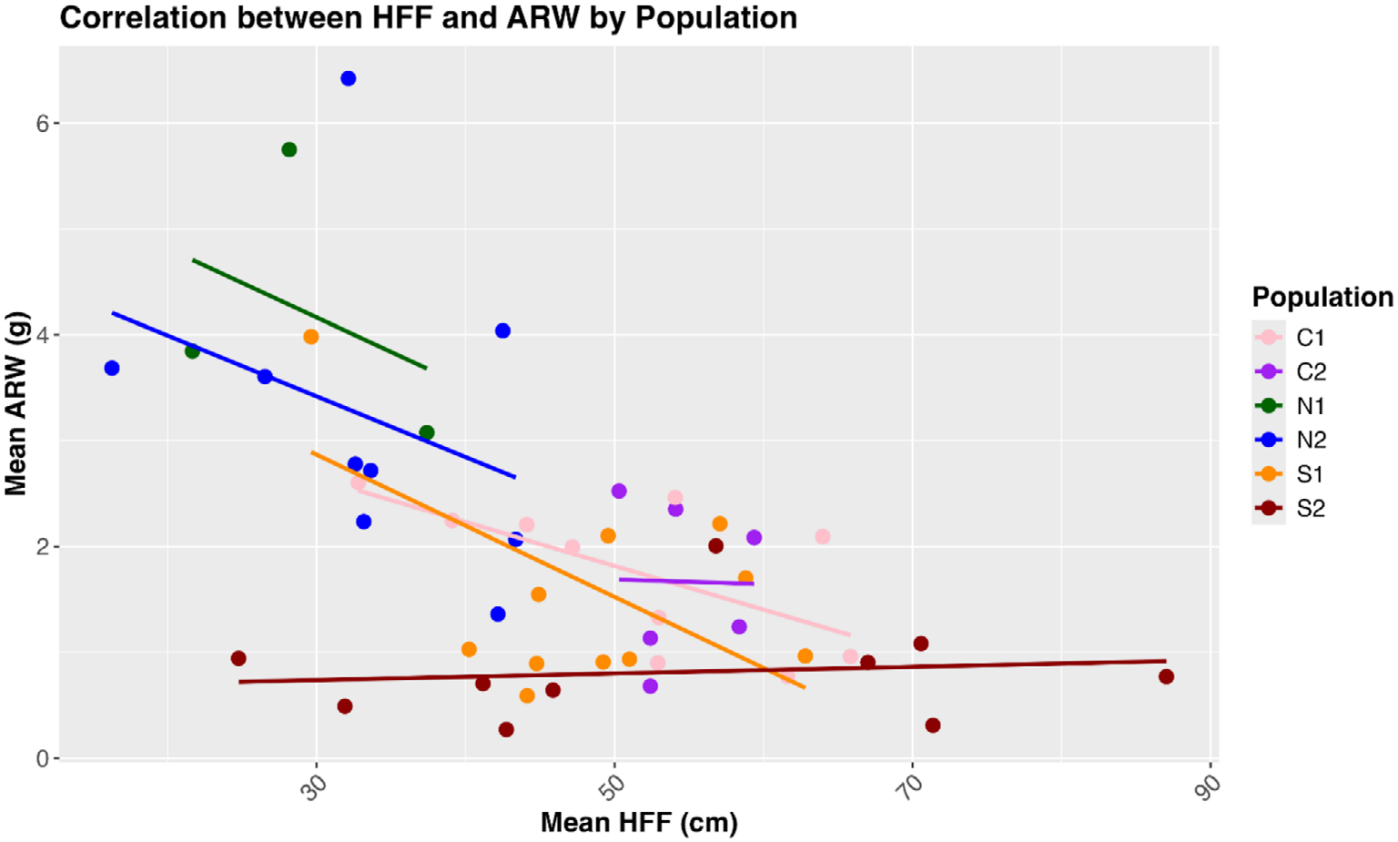
Correlation between height at first flower (HFF) and average rhizome weight (ARW) (*p* = 3.05e‐05). There is a strong negative correlation such that plants that are taller at first flower have less rhizome production. Pearson correlation coefficient = −0.554.

The linear mixed model showed that population and the interaction of population and year significantly affected height at first flower (HFF), while year alone did not (Table 8). The two northern populations had significantly lower HFF than the central (mean = −14.70 cm) and southern populations (mean = −16.02 cm) (Figure 8). On the other hand, central and southern populations were generally very similar to each other, except in C1 and S2, where S2 was about 3.3 ± 0.821 cm taller at first flower (*t* (1482) = 4.013, *p* = 0.0009). There was no regional pair that differed significantly. The only population to exhibit significant change in descendants relative to ancestors was S2, with an average reduction in height of 4.09 ± 1.12 cm (*t* (1293) = 3.64, *p* = 0.0017). The fitted linear models for rhizome data showed a significant effect of population on average rhizome weight (ARW) (*F*_5,44_ = 8.5244, *p* < 0.0001), a marginally significant effect on rhizome number (*F*_5,44_ = 2.3077, *p* = 0.06025), and no significant effect on total dry weight (*F*_5,44_ = 0.9705, *p* = 0.4463). In Tukey post hoc tests of total dry weight and rhizome number, there was no difference between any population pair, although there was a general trend of decreasing total dry weight (Figure 9) and increasing rhizome number (Figure 10) from south to north. For ARW, any pairing of northern and non‐northern populations is significantly different, except N2 and C2 (*p* = 0.064), with northern populations having three to four times greater ARW than lower latitude populations (Figure 11). Southern and central populations were generally the same, although S2 exhibited the lowest ARW, with an average weight of 0.8107 g. There was a strong negative correlation between HFF and ARW among populations (*r* = −0.554, *p* = 0.00003) (Figure 12).

**TABLE 8:** Summary statistics for linear mixed model of HFF (height at first flower).

Effect	Variance	SD	Marginal *R* ^2^	Conditional *R* ^2^	Sum sq.	Mean sq.	NumDF	DenDF	*F* value	*p*
Dam_ID	28.851	5.371	0.208	0.326	—	—	—	—	—	—
Residual	163.456	12.785	—	—	—	—	—	—	—	—
Population	—	—	—	—	112,775.215	22,555.043	5	1237.694	137.988	0.000
Year	—	—	—	—	319.598	319.598	1	1197.317	1.955	0.162
Population:Year	—	—	—	—	2081.882	416.376	5	1237.694	2.547	0.026

## 4 Discussion

### 4.1 Phenological Curve Metrics and FPCA (Objective 1)

Our study is unique in that we generated complete phenological curves for flowering phenology in *Erythranthe cardinalis* , allowing us to describe intraspecific variation in entire flowering seasons, rather than point estimates like day of first flower (Sheth and Angert 2016; Vtipil and Sheth 2020; Anstett et al. 2021; Nelson et al. 2021). By describing entire flowering curves through integration, we address a critical knowledge gap in understanding how early and late‐season flowering behavior co‐vary within populations and how this variation may respond to environmental change (Forrest and Miller‐Rushing 2010; CaraDonna et al. 2014). Our five phenological curve metrics and first Functional Principal Component indicated that with increasing latitude of origin, populations' curves begin, peak, and end earlier when grown at their trailing range edge. Interpopulation differences were most pronounced by the end of the flowering season, highlighting the adaptive significance of mechanisms increasing late‐season flowering in drought‐prone environments. Due to southern populations' historic persistence in extreme environments, they have likely evolved drought‐avoidant traits that allow them to extend flowering during harsh conditions (Kenney et al. 2014; Pironon et al. 2017). Yet descendants across populations reached Season End earlier, specifically in C1, suggesting that contemporary evolution is not reinforcing drought‐avoidant traits that extend late‐season flowering, pushing phenotypes further from southern populations', contrary to what might be expected to “keep pace” with climate change (Hickling et al. 2006).

The lack of change in descendants for early‐season metrics suggests populations are stable enough to resist multiyear perturbations in drought severity (Vtipil and Sheth 2020), although the ubiquitous directional shift toward an earlier end of season hints populations are approaching the limit of this stability under increasing drought severity (Jump and Peñuelas 2005). In addition, as *E. cardinalis* is a perennial plant, it is possible too few generations have passed between 2010 and 2017 to result in detectable changes in traits or allele frequencies. High variance in early season flowering across populations, as conveyed by PC2 and the random effect of sire on metrics, suggests this may have a buffering effect against selective pressure. Although insignificant, the trend of descendants beginning Season Start earlier in S2 but later in N1, causing them to become undifferentiable along PC2, implies trailing and leading populations may eventually experience stabilizing selection for early‐season flowering. Researchers commonly predict that the majority of phenological evolution occurring in a species manifests itself before peak flowering (D. W. Inouye 2008, 2022), often due to high genetic variance and heritability of time of first flower (Brachi et al. 2011). Although we found evidence for the latter, we only observed an evolutionary shift for late‐season flowering in C1 descendants. However, peak flowering seemed to be an inflection point that precipitated this shift, as shown through simultaneous change across both FPCs toward earlier flowering and an earlier‐trending Peak Center metric. We are among the first to use FPCA to analyze entire flowering curves (Tecuapetla‐Gómez et al. 2024) and demonstrate its potential to illuminate nuances that would otherwise go unnoticed. Previously, NDVI (Normalized Difference Vegetation Index) curves have been leveraged through FPCA to link autumn productivity to overwintering survival in deer (Hurley et al. 2014) and classify NDVI curves by plant associations with greater accuracy than geographic models (Pesaresi et al. 2020). Through this novel method, we reinforce the value of high phenotypic diversity as a buffer to selective pressure and propose that late‐season phenological evolution may precede the first signs of early‐season change. We find that contemporary evolutionary pressures may counteract phenotypes historically maintained by biogeographic influences, implying any spatially distributed taxa may evolve in novel ways under climate.

### 4.2 Genetic Variance at Trailing and Leading Range Edges (Objective 2)

Confidence intervals for heritability estimates ranged from 0.34 to 0.71, raising some concern about precision. This largely reflects methodological scaling of sire variance by four to obtain $V_{a}$, compounded by reduced sample size from concatenating individuals by dam to create half‐sibling sire families. Minor environmental variation in the garden, such as slope, soil compaction, or localized competition, could have influenced individual plants, but randomizing families and populations across garden blocks and concatenating full‐siblings further mitigated potential bias. Despite these factors, our results are robust and provide a reliable basis for interpreting genetic contributions to phenology.

Our results demonstrate substantial variation in the heritability of specific regions of flowering curves across populations, with strong genetic control over early‐season flowering and a greater environmental influence on late‐season flowering. This is biologically meaningful because naturally occurring populations are expected to harbor the greatest genetic diversity for alleles and traits that confer a fitness advantage (Barrett and Schluter 2008). On the other hand, the greater proportion of residual variance ($V_{R}$) in late‐season flowering metrics suggests that plasticity to environmental variation may play a larger role in shaping phenological responses later in the season. In Mediterranean climates like S1 with highly variable interannual moisture availability, plants that can opportunistically extend their flowering period late into the season might have higher $V_{a}$ from greater reproductive success, especially if pollinator availability remains high (Picó et al. 2002; Sánchez et al. 2012). During data collection, there was never a day when hummingbirds were not present (J. Waits, personal observation), so there was a period between August and September in which only southern plants were being pollinated.

We found the greatest heritability for Season Start in N2, followed by C1, followed by S1 and S2, which is opposite to what we expect for a *trailing‐leading* model of genetic diversity that predicts decreasing diversity from south to north (Pironon et al. 2017). However, considering each population as a component of a region introduces an alternative interpretation. In the south, both populations harbor a minimum heritability of 0.47 for Season Start, opposed to central and northern regions which each contain one population with heritability that is less than 0.26. These low points in heritability in C2 and N1 may represent reduced genetic diversity or high amounts of inbreeding, both of which could be results of frequent cloning via rhizomes or founder effects of a recent colonization event from the range core (Hampe and Petit 2005). Or, in the case of C1, which harbored the second highest heritability for earlier metrics but the lowest $h^{2}$ for Peak End and Season End, low points may reflect a reduction in diversity following evolutionary change or genetic drift. Through this lens, we find evidence for increased additive genetic variance and heritability in the south when we average populations by region, reflecting their shared origin from different range edges. This contention is further supported by S1 and S2 both having greater heritability for PC2 than C1, although still less than N2. This, combined with greater $V_{a}$ for Season End, would support a *trailing‐leading* model put forth by Hampe and Petit (2005) and Pironon et al. (2017) in which trailing populations have low within‐population diversity but high between‐population regional diversity due to ancient persistence through glaciation cycles. The peak in both $V_{a}$ and heritability for early‐season flowering in N2 could be explained by a prediction from Pennington et al. (2021) that cool edge populations can accumulate genetic diversity over time from gene flow from warmer populations, although this can be swamping or maladaptive. Given that N2 is also at a much higher elevation (914 m) than N1 (295 m), it could be relatively older and more stable through time. However, greatest genetic variance in the north does not align with the results of Paul et al. (2011), who found increasing genetic diversity from the northern edge towards the center. In summary, trailing populations of latitudinally distributed species likely do harbor substantial regional diversity for phenotypic traits (Sheth and Angert 2016), although this appears to buffer against evolutionary change in *E. cardinalis* rather than promote it.

### 4.3 Relationship Between Rhizomes and Phenology (Objective 3)

In this study, we found evidence for a latitudinal trade‐off between stem height and rhizome allocation when grown at the trailing edge, such that northern plants produce more rhizomes and are shorter at first flower than central and especially southern populations. The descendant cohort of S2 displaying a reduction in HFF aligns with records of it experiencing the greatest climatic moisture deficit of all six populations between 2010 and 2017 (Vtipil and Sheth 2020), corroborating a past finding that southern plants did not produce rhizomes and were adopting a drought escape strategy (Nelson et al. 2021). A nearly horizontal trend line for the relationship of stem height and rhizome allocation in S2 demonstrates there is no variation for rhizome production, and most resources are allocated towards roots or aboveground structures that promote water use efficiency (Seleiman et al. 2021). However, we found conflicting evidence regarding drought strategy when comparing across populations instead of years. Grown at their trailing range edge, northern populations exhibited the earliest flowering at the smallest size, indicative of drought escape, but exhibited enhanced rhizome allocation, which could be indicative of drought avoidance. This further highlights the temporal change in HFF seen in S2, as it was induced under natural conditions, rather than under extreme heat and drought stress in the case of northern populations, which ultimately experienced substantial end of year mortality (J. Waits, personal observation). Origin from a more mesic climate and low diversity has likely genetically fixed northern populations to produce rhizomes with little plastic response to extreme stress, causing them to adopt a drought “escape” strategy regarding phenology (Des Marais et al. 2013). This indicates the two strategies, clonal reproduction via rhizome and drought escape through phenology, are not mutually exclusive (Kooyers et al. 2014), although they clearly do not complement each other.

## 5 Conclusions

As suggested by Collins et al. (2021), analyzing the phenological curve over its entire distribution did not support a unison shift toward earlier flowering that is predicted by most researchers (Anderson et al. 2012; Petrauski et al. 2019). Instead, we found evidence for a species‐wide truncation in late‐season flowering, specifically in C1, and a loose trend of southern and northern populations respectively beginning their seasons earlier and later. If this trend grows stronger in the future, specifically at range edges, it is possible that climate change exerts a directional evolutionary force on late‐season flowering but a stabilizing force on early‐season flowering. The results of Anstett et al. (2021) corroborate our findings that climate change has unequal, if not opposite, effects on the evolution of *E. cardinalis* at different edges of its range, although they actually found that southern plants are evolving a later first flower date. A preliminary trend of a later season start in northern populations may reflect maladaptive evolution, as it is associated with a reduction in total reproductive output across the entire phenological curve (curve appears “pinched”).

Maladaptive evolution as a result of climate change has been found to be more common in populations from historically cooler and wetter environments (Anderson and Song 2020) which may also be experiencing swamping gene flow (Pennington et al. 2021), although future tests for fitness will need to confirm this in our system (Sheth et al., in review). Stabilizing selection may also be an optimistic sign for the persistence of the species, because directional selection has been found to decrease genetic diversity over multiple generations (Anderson et al. 2012), as could be the case for C1 in Season End. Stabilizing selection, on the other hand, is maintained and reinforced through gene flow between central and marginal populations, emphasizing the importance of maintaining connectivity between southern populations and the rest of the range, given high $V_{a}$ and heritability across metrics in the south (Davis and Shaw 2001; Carlson et al. 2014). Southern populations likely retain substantial evolutionary potential, allowing them to respond effectively to intensifying drought or shifting pollinator dynamics. This flexibility may enable multiple adaptive trajectories, including earlier flowering under warming conditions or the maintenance of phenological breadth in response to fluctuating climates. In addition, relative to each other, S2 and S1 demonstrated two seemingly contradictory strategies within a small geographic region: (1) earlier flowering with a temporal reduction in size and (2) no change in flowering with consistent rhizome production. On the other hand, the persistence of southern populations themselves likely depends on maintaining gene flow between other southern populations to circulate a variety of phenotypes through the region and into intermixing zones (Hampe and Petit 2005). Lastly, it may not be meaningful to assign different populations of *E. cardinalis* to either “drought escape” or “drought avoidance” because of the oftentimes contradictory nature of their phenological traits. Rather, perhaps populations' phenotypes are the sum of historic biogeographic conditions (latitudinal differences) and contemporary climate change (temporal differences), and sometimes these influences can contradict each other in direction and magnitude. In turn, space‐for‐time restoration strategies for assisted gene flow may have unintended consequences, as evolutionary shifts in phenotype in the south may not serve populations in the north in the same way.

## Author Contributions

**Jordan Waits:** conceptualization (lead), data curation (lead), formal analysis (lead), investigation (equal), methodology (equal), project administration (equal), resources (supporting), software (lead), visualization (lead), writing – original draft (lead), writing – review and editing (equal). **Nathanael Larson:** investigation (supporting), resources (supporting). **M. C. Moazed:** conceptualization (supporting), data curation (supporting), investigation (equal), methodology (equal), project administration (equal), resources (supporting). **Ashley Regan:** investigation (equal), resources (supporting), validation (equal). **Marissa Strebler:** investigation (equal), resources (supporting), validation (equal). **Lluvia Flores‐Rentería:** conceptualization (supporting), funding acquisition (lead), methodology (equal), project administration (equal), resources (lead), supervision (lead), writing – review and editing (equal).

## Funding

This work was supported by the Division of Environmental Biology (2131815, 2131817, 2131819).

## Conflicts of Interest

The authors declare no conflicts of interest.

## Supporting information


**Data S1.** 3_prunpropo

**Data S2.** 3_final_code_jw

## Data Availability

All of the required data and code are provided as [Link], [Link].
